# Surgical Complication of Omental Infarction in Ulcerative Colitis Following Laparoscopic Colectomy

**DOI:** 10.7759/cureus.76304

**Published:** 2024-12-24

**Authors:** Mena Louis, Nathaniel Grabill, Jerrell Fang, Daniel Sarmiento Garzon

**Affiliations:** 1 General Surgery, Northeast Georgia Medical Center Gainesville, Gainesville, USA; 2 Surgery, Northeast Georgia Medical Center Gainesville, Gainesville, USA; 3 Colorectal Surgery, Northeast Georgia Medical Center Braselton, Braselton, USA

**Keywords:** acute abdomen, ct imaging, diagnostic laparoscopy, hemoperitoneum, omental infarction, omentectomy, postoperative complications

## Abstract

Omental infarction is a rare cause of acute abdomen, often mimicking more common abdominal emergencies such as appendicitis and cholecystitis, presenting significant diagnostic challenges. A 47-year-old male with a history of ulcerative colitis underwent laparoscopic total colectomy with end ileostomy. Postoperatively, he developed severe abdominal pain, chills, nausea, and increased abdominal distension. Despite having output from his ileostomy, his symptoms persisted. A CT scan revealed free intraperitoneal air and significant intra-abdominal fluid, indicating potential intra-abdominal injury. Diagnostic laparoscopy identified an infarcted omentum and 850 mL of hemoperitoneum. An omentectomy was performed, and the patient received supportive care postoperatively, leading to gradual improvement in symptoms and recovery.

In this case, surgical intervention was required due to severe symptoms, diagnostic uncertainty, and associated hemoperitoneum. While conservative management has been described in stable cases, this approach was not appropriate for our patient. Advanced imaging techniques, particularly CT, remain crucial for identifying omental infarction, but clinical judgment and individual patient factors ultimately guide management decisions.

## Introduction

Omental infarction is a rare cause of acute abdominal pain, accounting for fewer than 1% of all cases [1,2]. This condition, which involves the necrosis of the omentum due to compromised blood supply, often presents with symptoms that mimic more common abdominal emergencies such as appendicitis, cholecystitis, and diverticulitis [3]. Diagnosing omental infarction can be difficult due to its vague clinical symptoms and the fact that it is rarely considered a primary diagnosis by surgeons [4,5].

First described in 1899, omental infarction can be classified into primary and secondary types [6]. Primary omental infarction occurs spontaneously without any apparent underlying cause, while secondary omental infarction is associated with factors such as abdominal surgery, trauma, or hypercoagulable states [7​]. The right side of the omentum is more commonly affected due to its limited blood supply compared to the left side, which has a richer vascular network​ [7,8].

Imaging, particularly CT, is crucial in diagnosing omental infarction [9]. CT findings typically include a well-circumscribed, heterogeneous fatty mass with surrounding inflammatory changes and possible free fluid in the peritoneal cavity [9,10]. These radiographic features aid in differentiating omental infarction from other acute abdominal conditions, thereby guiding appropriate management strategies [10​].

The management of omental infarction can be either conservative or surgical, depending on the severity of symptoms and the presence of complications [11,12]. Conservative management involves supportive care with analgesics and anti-inflammatory medications, while surgical intervention, such as laparoscopic omentectomy, is reserved for cases with severe symptoms or diagnostic uncertainty [12​]. The choice of treatment approach should be individualized based on the patient’s clinical condition and response to initial management [13,14].

## Case presentation

A 47-year-old male with a long-standing history of ulcerative colitis presented with persistent gastrointestinal symptoms, including cramping abdominal pain, diarrhea, hematochezia, and mucous stools. Despite multiple therapeutic attempts with medications such as infliximab (Remicade), adalimumab (Humira), vedolizumab (Entyvio), upadacitinib (Rinvoq), and steroids, he showed minimal improvement. A recent colonoscopy had revealed active proctocolitis, leading to the recommendation for surgical intervention.

The patient underwent a laparoscopic total colectomy with end ileostomy. The surgery was uneventful, and he was discharged in stable condition with good pain control and dietary tolerance. However, upon returning home, he developed severe abdominal pain, chills, nausea, and increased abdominal distention despite having output from his ileostomy.

**Figure 1:**
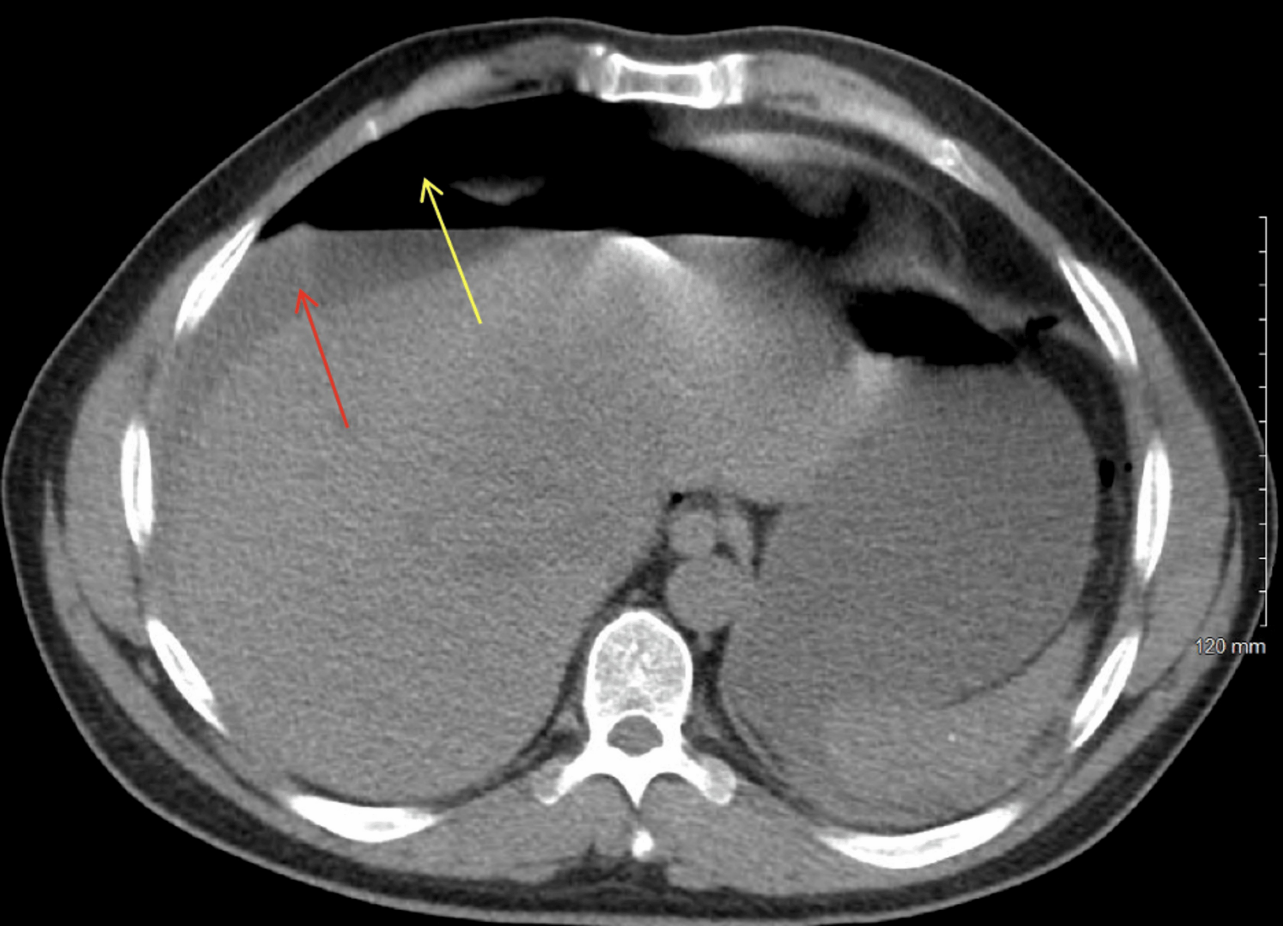
CT scan of the abdomen and pelvis without contrast (axial view) demonstrating a substantial amount of free intraperitoneal fluid (indicated by the red arrow) and extensive pneumoperitoneum (indicated by the yellow arrow). These findings are highly concerning for a perforated viscus.

**Figure 2:**
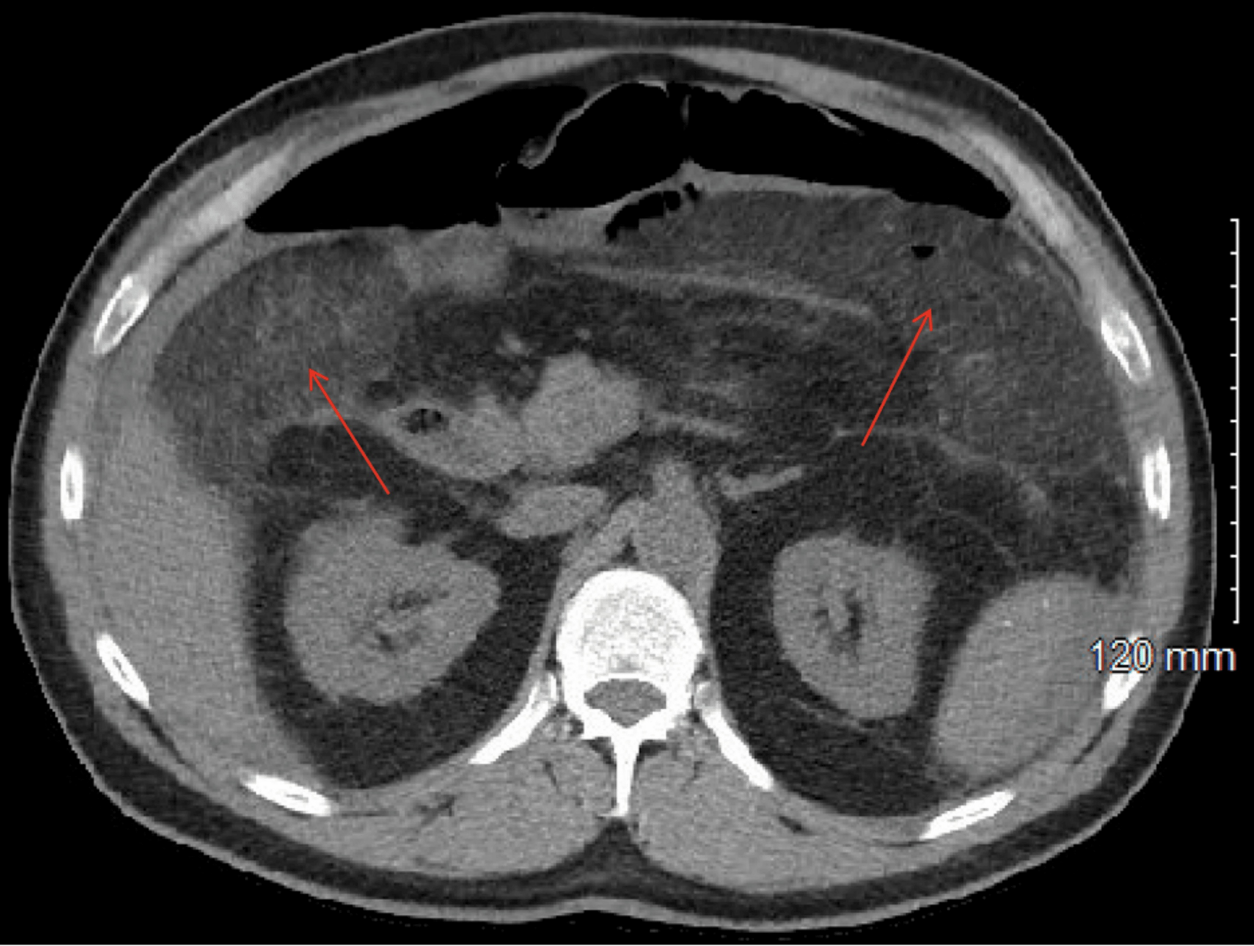
CT scan of the abdomen and pelvis without contrast (axial view), highlighting notable postoperative findings. The image reveals mesenteric stranding, which is possibly postoperative in nature (indicated by the red arrows). This mesenteric stranding may suggest inflammatory or ischemic changes related to the recent surgical intervention.

**Figure 3:**
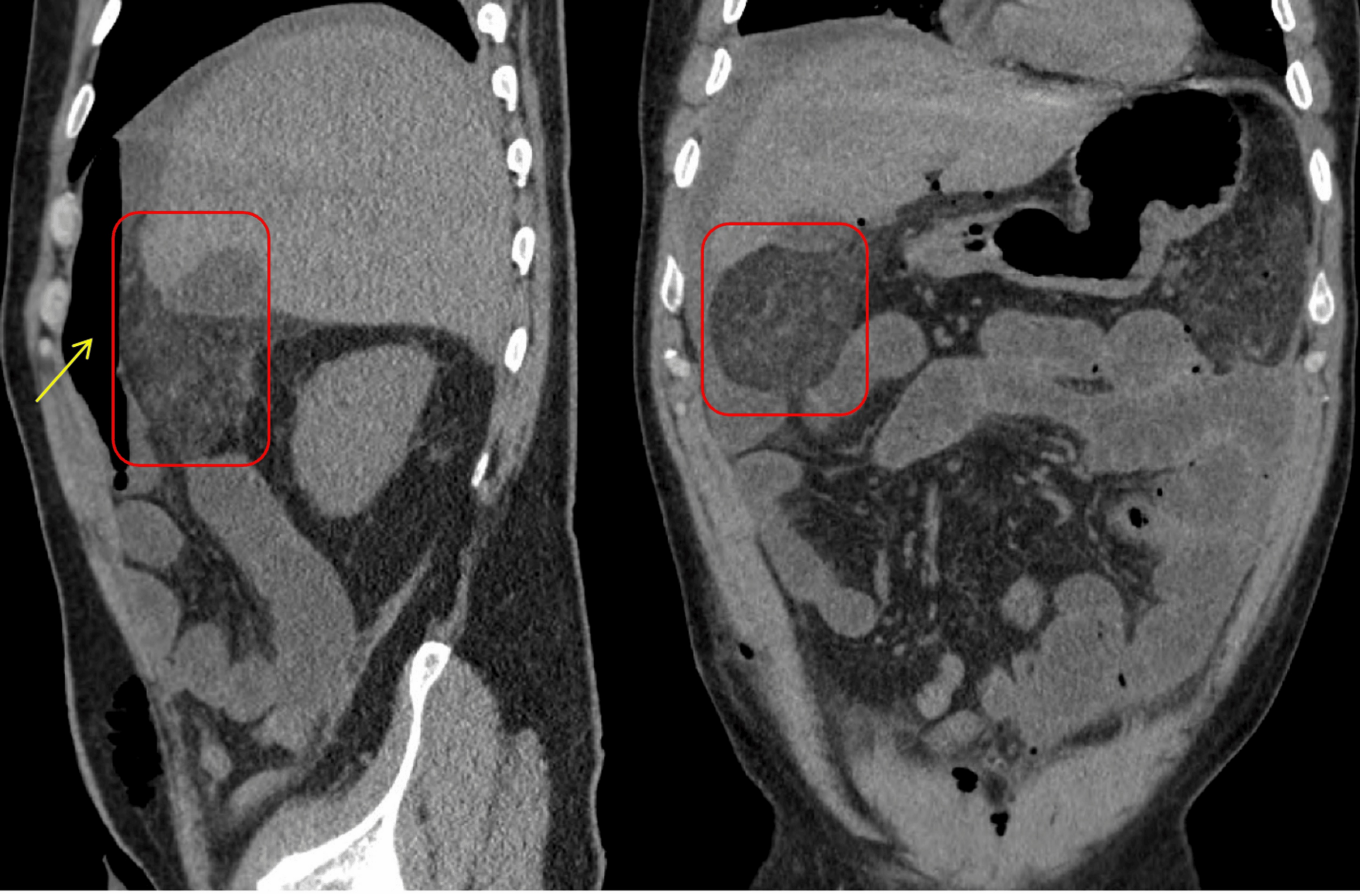
CT scans of the abdomen and pelvis without contrast presented in sagittal (left) and coronal (right) views. The images reveal mesenteric stranding and the presence of pneumoperitoneum (highlighted by the yellow arrow) in the right upper quadrant. The red boxes delineate the areas of mesenteric stranding, suggesting possible postoperative changes or complications.

A CT scan of the abdomen and pelvis showed free intraperitoneal air and a significant amount of intra-abdominal fluid, indicating a potential intra-abdominal injury (Figures 1-3). Given his peritoneal signs and worsening leukocytosis, the decision was made to proceed with diagnostic laparoscopy.

Diagnostic laparoscopy revealed 850 mL of hemoperitoneum and an infarcted omentum. An omentectomy was performed, and the patient received supportive care postoperatively. His recovery was marked by gradual improvement in symptoms, with stable ostomy output and resolution of nausea and vomiting.

The patient’s follow-up showed continued improvement, with plans for future surgical interventions, such as a completion proctectomy or proctectomy with ileoanal pouch anastomosis, discussed based on his clinical progress.

## Discussion

In this case, the initial suspicion of a perforated viscus was based on CT findings of free intraperitoneal air and significant intra-abdominal fluid, often associated with gastrointestinal perforation. However, the imaging did not reveal key features such as extraluminal contrast or bowel wall disruption typically seen in perforation. Therefore, diagnostic laparoscopy was performed to clarify the source of these findings. The intraoperative evaluation identified an infarcted omentum and hemoperitoneum, ruling out a perforated viscus and confirming the diagnosis of omental infarction. While the CT findings raised concerns for intra-abdominal pathology, they were not specific enough to establish the diagnosis of omental infarction preoperatively. Surgical intervention was essential to address hemoperitoneum and confirm the diagnosis.

Omental infarction is a rare cause of acute abdomen that can present significant diagnostic challenges due to its non-specific clinical symptoms and its ability to mimic more common abdominal pathologies such as appendicitis, cholecystitis, and diverticulitis [2]. This condition involves the necrosis of the omentum due to compromised blood supply, which can occur spontaneously or secondary to surgical procedures, trauma, or hypercoagulable states [14].

Omental infarction results from the interruption of the omental blood supply, leading to ischemia and subsequent necrosis [15]. It can be classified into primary and secondary types [16]. Primary omental infarction occurs without an apparent cause and is thought to be related to anatomical variations, such as a longer omental segment or abnormal vascularity [17]. Secondary omental infarction is associated with conditions that increase intra-abdominal pressure or cause direct trauma to the omentum, such as previous abdominal surgery, trauma, or underlying hypercoagulable states [18].

The omentum, an apron-like fold of peritoneum extending from the stomach and proximal part of the duodenum to adjacent abdominal organs, has a rich and complex vascular network [19,20]. The greater omentum is primarily supplied by the right and left gastroepiploic arteries, branches of the gastroduodenal and splenic arteries, respectively [21]. These arteries form a vascular arc along the greater curvature of the stomach, providing multiple anastomoses that ensure adequate blood flow [21]. Additionally, the lesser omentum receives blood from the right and left gastric arteries, which supply the lesser curvature of the stomach. Despite this extensive network of vessels, the omentum is susceptible to ischemia and infarction if the blood supply is compromised, such as through torsion, embolism, or surgical trauma [22].

The right side of the omentum is more commonly affected due to its relative anatomical mobility and longer length, which makes it more susceptible to torsion and subsequent infarction [23]. Patients typically present with acute abdominal pain localized to the right lower quadrant, although pain can also occur in the epigastric or left abdominal regions [24].

Omental infarction is often not considered in the initial differential diagnosis of acute abdomen [13,25]. Imaging, particularly CT, is crucial in identifying omental infarction [26,27]. Typical CT findings include a well-circumscribed, heterogeneous fatty mass with surrounding inflammatory changes and possible free peritoneal fluid [27]. These imaging features help differentiate omental infarction from other acute abdominal conditions, guiding appropriate management strategies [28].

Management of omental infarction can be either conservative or surgical [28]. Conservative treatment includes pain management, anti-inflammatory medications, and close clinical monitoring [29]. This approach is often sufficient as omental infarction can resolve spontaneously [30,31]. A study by Medina-Gallardo et al. (2020) supported the effectiveness of conservative treatment, highlighting that many cases are resolved without surgical intervention [30]. However, surgical intervention, typically via laparoscopic omentectomy, may be necessary in cases with severe symptoms, diagnostic uncertainty, or complications such as peritonitis or abscess formation [31]. Other potential complications of omental infarction include bowel obstruction, abscess formation, and sepsis. These severe outcomes typically result from inflammation, secondary infection, or compression of adjacent structures.

## Conclusions

Omental infarction has a favorable prognosis when promptly diagnosed and managed, with most patients achieving full recovery. While CT imaging is invaluable for identifying potential intra-abdominal pathologies, surgical exploration remains crucial in cases of diagnostic uncertainty or complications. Timely intervention can prevent severe outcomes such as bowel obstruction, abscess formation, or sepsis.
